# Bovine tuberculosis (TB) in herds with long-duration of official freedom during a period of national resurgence of infections

**DOI:** 10.1186/s13620-026-00330-w

**Published:** 2026-01-21

**Authors:** Michael Horan, Damien Barrett, Sam Smyth, Andrew W. Byrne

**Affiliations:** 1 Department of Agriculture, Food and the Marine, Ruminant Animal Health Division, Backweston, Dublin, Ireland; 2 School of Veterinary Medicine, University College Dublin, Belfield, Dublin 4, Ireland; 3 Department of Agriculture, Food and the Marine, One Health Scientific Support Unit, Agriculture House, Dublin, Ireland

**Keywords:** Zoonotic TB, Badger vaccination areas, Veterinary epidemiology, Risk factors, BTB freedom

## Abstract

Herds which experience long periods of bTB freedom enjoy lower prospective breakdown risk. However, during periods of rising infection levels, as seen in Ireland in recent years, the number of breakdowns in low-risk herds can also increase. In this context, an analysis was undertaken to investigate the breakdown risk of long-term officially TB-free herds (≥ 10 years without a breakdown, “C10” herds), during 2016–2024 in Ireland. Furthermore, we performed a cohort study using a mixed effects logistic regression model to explore the correlates of breakdown probability for C10 herds over an 18-month risk period. The length of time a herd had been officially free was associated with a decline in the risk of breakdown during the at-risk period from January 2023 to June 2024; for example, 17.1% of herds officially free for less than 1-year (C0) had a breakdown during the at-risk period, relative to 4.6% of C10 herds. C10 herds had greater risk of breakdown during the risk period if they were a dairy (OR: 1.806; 95%CI:1.599–2.041; *P* < 0.001) or drystock herd (OR: 1.242;; 95%CI:1.092–1.413; *P* < 0.001), relative to breeding non-dairy herds; had larger herd size (OR: 1.547 for each additional unit of log_e_-herdsize; 95%CI: 1.475–1.622; *P* < 0.001); if they had any evidence of a breakdown in the past (beyond 10 years) (inverse OR: 1.348; 95%CI: 1.245–1.458; *P* < 0.001), and with increasing inward movements of animals (e.g. ≥ 90 vs. < 30 moves, OR: 2.395 (95%CI:1.881–3.049;*P* < 0.001). However, the effect of inward movement varied by herd-type, with movements being more important in non-dairy herds. There was no evidence to suggest that C10 herds in areas (district electoral divisions) wholly or partially designated for badger vaccination were at increased, or decreased, risk of breakdown relative to areas not designated for badger vaccination (*P* > 0.96). These data provide additional evidence of the importance of bTB history as a risk factor for bTB breakdown.

**Supplementary Information:**

The online version contains supplementary material available at 10.1186/s13620-026-00330-w.

## Introduction

Animal tuberculosis is caused by species of *Mycobacterium* within the *M. tuberculosis* complex, and includes bovine tuberculosis (bTB), predominantly caused by *M. bovis.* bTB continues to be an endemic infection of cattle in Ireland despite several decades of control efforts as part of an integrated national programme [1]. The long-term maintenance of bTB is associated with a complex range of interconnected factors related to the limitations of diagnostic tests available, structure and connectivity (via trade links and spatial contiguity) of herds, recrudescence within herds, and the presence of a widespread and abundant wildlife host, the European badger (*Meles meles*) [2]. Badgers have been managed in Ireland via targeted culling at a national level since 2004, with the intention of reducing badger abundance significantly in local areas where bTB outbreaks occur in cattle [3]. Since 2018, based on experimental and field trial data [4–6], a policy of badger vaccination has also been part of the national control programme. The numbers of badgers vaccinated increased steadily to over 40% of all captures by 2021 [7], across a designated area of 20,000km^2^ representing over half the total area under capture [1]. While wildlife interventions are generally supported by farmer groups and stakeholders [8], several studies suggest there is less support for vaccination relative to culling based on perceptions of efficacy (see for a review: [9]).

Despite the challenges of bTB control, the vast majority of herds in Ireland at a given time are officially TB free and are allowed to trade. The longer herds remain TB free, the lower their risk of breakdown in the future. This has been reflected in research from Ireland and elsewhere, where a history of bTB infection has been found to be an important risk factor for breakdowns (e.g. [10, 11]). Since 2020, bovine herds in Ireland have a bTB risk status (TB Herd History Risk (TBHHR)) — corresponding to the length of time they have been free of disease [1]. This varies from currently infected “INF”, free for a varying number of full years from 0 to 9 (“C0” to “C9”), to free for ten or more full years (“C10”). Based on research, C10 herds have long been regarded as having a very low risk of future breakdown compared to other herds [12]. It should be noted that the TBHHR status is affected primarily by how much time has elapsed since a herd was last in a breakdown, but will also be affected by how long a herd has been established in the case of recently established herds.

Ireland is currently experiencing a resurgence in bTB, with substantial increases at both herd and animal level over the past decade. The national herd incidence was at a record low level of 3.27% for 2016, and the number of reactors was 16,914. These numbers had increased substantially up to 2022, when the herd incidence and the total number of reactors had reached 4.31% and 23,393, respectively. That increase in key metrics accelerated sharply from 2022 to 2024. Final figures for 2024 indicate a herd incidence of 6.04% and 41,682 reactors, with 6,254 herds newly restricted over the course of 2024 (provided by Ruminant Animal Health Policy Division, DAFM, 30th September 2025). In Ireland, reactor animals per thousand tested on the skin test is generally used to indicate the burden of disease at animal level. The skin test APT was 2.95 in 2024, also markedly increased from its 2022 level of 1.79. The particularly sharp increase in key TB metrics from 2022 to 2024 is driving a vigorous debate regarding the future of the programme and its aims of eradication [13].

Ireland implements a very comprehensive programme to control the spread of bTB. Each bovine herd has all bovine animals present tested at least once per year, with only bovines aged less than six weeks at the time of the test exempt. Additional skin and/or blood tests are scheduled for herds in response to increased neighbourhood risk, tracebacks or to resolve the status of herds in breakdown, so that many herds (and animals) will be tested multiple times within the same year.

Despite the extensive measures already being taken to counter bTB, it is generally accepted that additional control measures need to be adopted for Ireland to reverse the recent increase in bTB, and that any control measures selected should be informed by the available evidence. By mid-2024, some stakeholders were of the opinion that the surge in bTB seen from early 2023 up to that point was associated with increasing numbers of herds with long-term freedom experiencing breakdowns. Additionally, it has been hypothesised that a high proportion of these bTB breakdowns are found in badger vaccination areas (relative to non-vaccine areas).

To explore these hypotheses, our objectives were to:

1. Evaluate the trends in per annum probability of C10 herds experiencing a breakdown between 2016 and 2024 to assess whether there was an increasing relative risk of breakdown for this cohort over that period.
2. Explore risk factors associated with a breakdown during an 18-month risk period for C10 herds; in particular, whether a herd being based in an area designated for badger vaccination was a risk factor for bTB breakdown.

The study was commenced in July 2024, with a particular focus on a recent upsurge in bTB levels. The study period from 2016 onward was selected as it allowed us to describe the pattern in the risk of C10 herds experiencing breakdown as Ireland moved from the lowest recorded national herd incidence of 3.3% in 2016 to far higher levels of herd incidence in recent years. The 18-month period from 1^st^ of January 2023 to 30^th^ of June 2024 was chosen as the risk period as it was recognised that recorded levels of bTB had increased markedly during this period relative to the years immediately preceding it. Therefore, it was believed that an analysis using this 18-month timeframe as the risk period would be most informative to understand the recent surge, which was of great concern to policymakers and stakeholders alike.

## Methods

### Data sources and study design

Datasets from DAFM’s Animal Health Computer System (AHCS) were collated and exported via CSV files for further processing and analysis. Movement data were gathered from the Animal Identification and Movement (AIM) database. TBHHR data from 1 st January 2016 to 30th June 2024 were examined as part of the investigation. Detailed descriptive analysis was undertaken. The TBHHR status for all herds for a given year was retrieved, ranging from INF (currently restricted), C0 (less than one year since a breakdown), C1 (at least one year but less than two years since a breakdown), through to C10 (10 years or more since a breakdown). An additional category “D” is attributed to dealer herds because there was a consensus among policymakers that the time since last breakdown does not fully reflect the disease risks arising from the high frequency of movements into, and out of, these herds. TBHHR was applied to most herds for the first time during 2020. Accordingly, the TBHHR was estimated for herds at timepoints when no TBHHR was on record. The estimation procedure was verified against known TBHHR statuses available for timepoints from 2021 onward, and produced almost identical counts of C10 and non-C10 herds as the actual TBHHR designation on AHCS.

### Classification of herds according to local bTB wildlife policy

The potential risk associated with local bTB wildlife policy was derived by associating each herd with the District Electoral Division (DED) within which it resided. District Electoral Divisions (DEDs) are small areas used for administrative purposes in Ireland. There are 3,420 DEDs in Ireland with DEDs having a typical size of 20km^2^. As of 2024, there were 2,833 such DEDs with cattle presented for testing in Ireland.

**Fig. 1:**
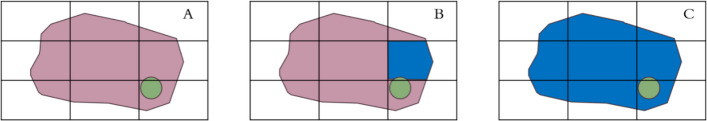
Schematic diagram of how herds (green dot) were designated a badger vaccination exposure depending on whether there were no vaccine quartiles (blue polygon) overlapping with district electoral divisions (DED) (pink polygon). **A** No badger vaccine in DED; **B** partial vaccination in DED; **C** total vaccination in DED

Control of bTB in wildlife in Ireland is based on a grid system comprising *quartiles*, which are 2 km × 1.5km rectangles. This grid system was used to assign each of these DEDs to a status of “no vaccine”, “partial vaccine” or “total vaccine” (see schematic in Fig. 1). We defined the ‘vaccination’ exposure as, the degree to which the DED on which the herd number is based is assigned to vaccination, as determined by inspection of a map of approximately 2,833 DEDs:

1. No vaccine—none of the land area of the DED is covered by vaccination quartiles (Fig. 1A)
2. Partial vaccine— some, but not all, of the land area in the DED is covered by vaccination quartiles (Fig. 1B)
3. Full vaccine— All of the land area in the DED is covered by vaccination quartiles (Fig. 1C).

These designations reflected the local policy applying to each DED. The actual coverage of vaccination achieved, or the efficacy of culling efforts, were not measured as part of this study.

### Statistical analyses

As a first step, the proportion of all breakdowns per year per TBHHR status was calculated to assess whether C10 status herds were increasing in their relative probability of breakdown over time. A simple linear model was used to assess the trend in the average proportion of all breakdowns that were in C10 herds over the time series.

Secondly, to assess the future risk of breakdown for herds based on their TBHHR status, a retrospective cohort approach was developed. Herd TBHHR status on the 31 st of December 2022 was used as a cut point, and the future probability of breakdown during an 18-month risk period was calculated. The 18-month risk period was chosen as being most representative of the bTB “surge”, characterised by dramatically increasing reactor counts, which was causing very substantial concern in summer 2024. Regarding the total herds at risk on 31 st of December 2022, all herds listed as ‘active’ on AHCS were included, except for research herds or any herds that had no AIM records for the period: i.e. no cattle on the 31 st December 2022 and no movements (of any kind) or births recorded during the previous twelve months. Two descriptive metrics were calculated, the proportion of herds breaking down attributable to each TBHHR status (herd-level metric) and the breakdown herds per 1000 animals (animal-level metric). The relationship between herd-level risk and area-level ‘exposure’ to either badger vaccination, partial badger vaccination or other was assessed (see above for a description of these exposures).

### Models

Multivariable models were used to investigate the risk of breakdown for herds which had a C10 status on the 31 st of December 2022. The outcome variable of interest was a binary response representing a herd having experienced a “breakdown” during a risk period from January 2023 to June 2024. A breakdown was defined as a period of trade restriction imposed on a herd that begins when one or more animals are disclosed as a bTB case, as determined by the skin test, interferon-gamma testing, or by the detection of visible lesions at routine slaughter which are then confirmed with *M. bovis* in the laboratory. The binary outcome was modelled using a logistic regression.

As it is known that bTB can be affected by spatial effects which can include administrative differences across geographies, a random effect was fitted for “county” effects. There are 26 counties within Ireland, with different counties having different levels of bTB risk. For comparative purposes, county was also fitted as a fixed effect in an alternate model.

Mixed effects multivariable logistic models were built using several independent variables including herd size (number of cattle), whether a herd had any history of a breakdown or not (> 10 years before present), inward movement histories, herd type and the policy for bTB control in wildlife in the local area. Herds were designated to one of three herd types using a simple classification system as follows. Herds were designated as “Dairy” if there was a milk supply contract (MSC) recorded on the DAFM’s AHCS for the herd on the 31 st of December 2022. Herds were designated as “Breeding non-dairy” if there was no MSC on DAFM records and the herd had at least one birth recorded during 2022. Herds were designated as “Drystock” if they had no MSC on the record and had zero births recorded in 2022.

All independent variables were offered to the multivariable model. Explorations of pairwise correlations between predictors was undertaken. First order interaction terms were explored between herd type and badger vaccination exposure, to explore whether vaccination exposure in dairy herds had different effects than in non-dairy herds. This was performed to explore the hypothesis that dairy herds were at increased risk of breakdown where areas were assigned to badger vaccination, relative to non-badger vaccine areas.

Furthermore, interactions between herd type and movement were also explored.

A likelihood ratio test was used to confirm that the county random effect improved the model fit, by testing whether ρ, the intraclass correlation coefficient, was equal to zero (meaning that the random effect was adding little to explaining the model). Competing models were compared using Akaike’s information criteria (AIC), with models with lower AIC considered best fitting models. Note, competing models only compared models including first order interactions, as baseline predictors were fitted to all models. The DED vaccination status was forced into all models, irrespective of association with the outcome, as this was a key variable of interest in the study.

Analyses were undertaken in R (version 4.4.1) and Stata 16 (IC).

## Results

### Changes in numbers of breakdowns in C10 herds over time (2016–2024)

Thirty-seven thousand six hundred eighteen breakdowns were recorded with their associated TBHHR status over the study period (2016–2024). 15,733 of the herds involved had C10 status immediately prior to the start of their respective breakdowns. The average proportion of all breakdowns that were in C10 herds has not increased over the time series (linear change: β = 0.002; R^2^ = 0.07; *p* = 0.48; Table 1). Over the study period, the proportion of herds which had C10 status at the start of a calendar year and broke down over that year averaged 2.8% (Table 1). The figures in Table 1 suggest that breakdowns in C10 herds did not take on a disproportionate role in the substantial increase in bTB levels from 1 st of January 2023 onward. However, there appeared an increase in the number of non-C10 herd breakdowns (β = 115 breakdowns per annum (2016–2023); R^2^ = 0.83), and the proportion of non-C10 herds breakdown (β = 0.4% increase per annum (2016–2023); R^2^ = 0.91).

**Table 1:** Total breakdowns, C10 breakdowns, and non-C10 breakdowns descriptive statistics by year, 2021- 2024

Column	A	B	C	D	E	F	G	H	I	J
Year	All herds start year	Total bTB breakdowns	BD/herd (B/A)	C10 herds at start of year	BD in C10 herds	C10 BD as % of C10 herds (E/D)	C10 BD as % of all breakdowns (E/B)	Non-C10 herds at start of year	BD in non-C10 herds	Non-C10 BD as % of non-C10 herds (I/H)
2016	113,513	3,685	3.25%	64,055	1,429	2.23%	38.78%	49,458	2,256	4.56%
2017	112,551	3,889	3.46%	64,617	1,612	2.49%	41.45%	47,934	2,277	4.75%
2018	111,317	3,888	3.49%	65,087	1,613	2.48%	41.49%	46,230	2,275	4.92%
2019	109,291	4,091	3.74%	65,675	1,748	2.66%	42.73%	43,616	2,343	5.37%
2020	108,028	4,814	4.46%	66,247	2,122	3.20%	44.08%	41,781	2,692	6.44%
2021	107,578	4,715	4.38%	66,113	2,031	3.07%	43.08%	41,465	2,684	6.47%
2022	107,523	4,632	4.31%	66,058	1,964	2.97%	42.40%	41,465	2,668	6.43%
2023	106,467	5,243	4.92%	65,910	2,110	3.20%	40.24%	40,557	3,133	7.72%
2024*	104,759	2,661	2.54%	64,811	1,104	1.70%	41.49%	39,948	1,557	3.90%
Total		37,618			15,733				21,885	
MH/12mth	109,003	4,426	4.06%	65,397	1,851	2.83%	41.82%	43,606	2,575	5.90%
MH without 24	109,534	4,370	3.99%	65,470	1,829	2.79%	41.85%	44,063	2,541	5.77%

^*^First 6 months of the year only

### Risk of breakdown in C10 herds versus other herds—descriptive results

We identified 106,467 active bovine herds on the 31/12/2022 from which two descriptive metrics of relative probability of breakdown (proportion with a breakdown and breakdown herds per 1000 animals) during an 18-month risk window could be calculated (Table 2).

**Table 2:** Risk of TB breakdown in the next 18 months (01/01/2023 to 30/6/2024) for herds with different TBHHR categories on 31/12/2022

*TBHHR categories*	*Herds n*	*Herds with* ≥ *1 breakdown*	*Animals in residence on 31/12/22*	*Prop. Herds with a breakdown*	*Breakdown herds per 1000 animals*
*A)*	(B)	(C)	(D)	= C/B	= 1000* C/D
*INF*	2718	534	423,691	0.197	1.260
*C0*	5411	924	503,671	0.171	1.835
*C1*	5501	648	423,895	0.118	1.529
*C2*	4637	486	327,839	0.105	1.482
*C3*	3583	368	264,852	0.103	1.390
*C4*	3157	294	215,404	0.093	1.365
*C5*	2923	247	203,797	0.085	1.212
*C6*	2933	221	198,275	0.075	1.115
*C7*	3488	255	215,594	0.073	1.183
*C8*	2961	202	195,844	0.068	1.031
*C9*	2969	203	187,439	0.068	1.083
*C10*	65,910	3049	3,298,508	0.046	0.924
*D*	276	10	14,965	0.036	0.668
*ALL*	106,467	7441	6,473,774	0.070	1.149

As small herds tend to have low risk, even if it could be shown that the risk per herd was decreased for C10 herds relative to other herds, the possibility remained that animals in C10 herds on 31/12/2022 were as likely, or more likely, to experience a breakdown than animals in other herds. Accordingly, the “breakdown herds per thousand animals” statistic was calculated to show the risk of experiencing a prospective breakdown at animal level.

Overall, the figures show that between 1st of January 2023 and 30th of June 2024, the risk of breakdown per herd and per animal declines with the length of time which a herd has been free of bTB, with C10 herds (4.6% breakdown; 0.9 breakdowns per 1000 animals; Table 1) continuing to have the lowest risk of regular commercial herds (i.e. excluding dealer or “D” herds). Herds with an infected status (“INF”, 19.7% herds) or free for less than 1 full year (“C0”, 17.1% herds) had the highest probability of breaking down during the risk period. The at-risk period for INF herds is limited by their status of being in a breakdown at the start of the risk period, so the breakdown risk shown in the table underestimates the risk for these herds to some degree.

### Herds exposure to badger vaccination in local area

Of the 106,467 cohort herds, 47,048 (44%) herds had no area allocated to badger vaccination in their DED, 40,655 (38%) herds had partial exposure to badger vaccination at DED level, and the remaining 18,764 (18%) herds, were assigned to DEDs which were fully allocated to vaccination. Visual checks of the distributions of herds size, herd type, and pre-existing TB risk as indicated by TBHHR suggested that there were no large differences for these attributes amongst the different vaccination exposure types (supplementary material Figures S1-S3). However, close examination of the herd types indicates a slightly greater proportion of drystock herds and a lesser proportion of breeding non-dairy herds in full-vaccine DEDs compared to non-vaccine areas. Similarly, a closer examination of the distribution of herd size shows a lower density of smaller herds in the 0–25 category in full-vaccine DEDs relative to non-vaccine DEDs. This difference in distribution of herd size is also apparent when only C10 herds are considered with the distribution being more right-skewed for full-vaccine areas relative to non-vaccine areas (Figure S4 and Table S1).

There were 65,910 herds with C10 status which met our inclusion criteria on 31/12/2022; 3,049 (4.63%; Table 3) of these went on to have breakdowns during the 18-month study period. The proportion of C10 herds experiencing a breakdown in full vaccine DEDs (5.16%) was higher relative to partial (4.66%) and non-vaccine areas (4.38%; Table 3).

**Table 3:** C10 herd breakdowns by DED badger intervention status during the 18-month study follow-up period

	*Total C10 herds*	*Herds with* ≥ *1 breakdown (%)*	
*Non vaccine DED*	28,764	1,260	*(4.38%)*
*Part vaccine DED*	25,449	1,186	*(4.66%)*
*Full vaccine DED*	11,697	603	*(5.16%)*
*All C10 herds (total)*	65,910	3,049	*(4.63%)*

### Model outputs

The random effects final model had 65,910 observations, with 3,049 breakdowns (4.6%), modelled across 26 counties with a minimum, mean and maximum, of 142, 2,535, and 7,344 observations per county, respectively. The final model significantly explained variation in the outcome relative to a null model (χ^2^ = 1290, *p* < 0.001; Table S2). There was evidence to suggest that the random effect (RE) improved the fit of the model (test of ρ = 0 (χ degrees of freedom = 1) = 129, *p* < 0.001; see figure S5 for normal plot of county-level residuals), relative to a model without the county RE. This variance amongst counties level (*n* = 26) was reflected in the marginal predicted probability of breakdown, from the fixed effect model (Table S3; Fig S6). One tested interaction was significant (herd-type*inward movements) and retained in the final multivariable model. The parameter estimates for the full model is presented in the supplementary material (Table S2).

**Fig. 2:**
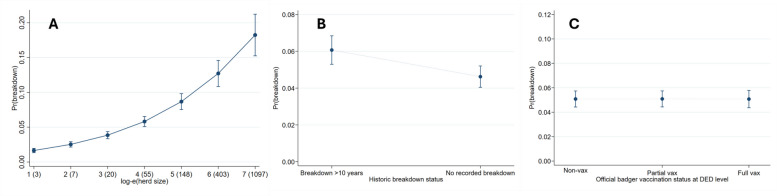
Predicted margins between breakdown risk in C10 herds and (**A**) log herd size, (**B**) historic infection, and (**C**) local area vaccination treatment

**Fig. 3:**
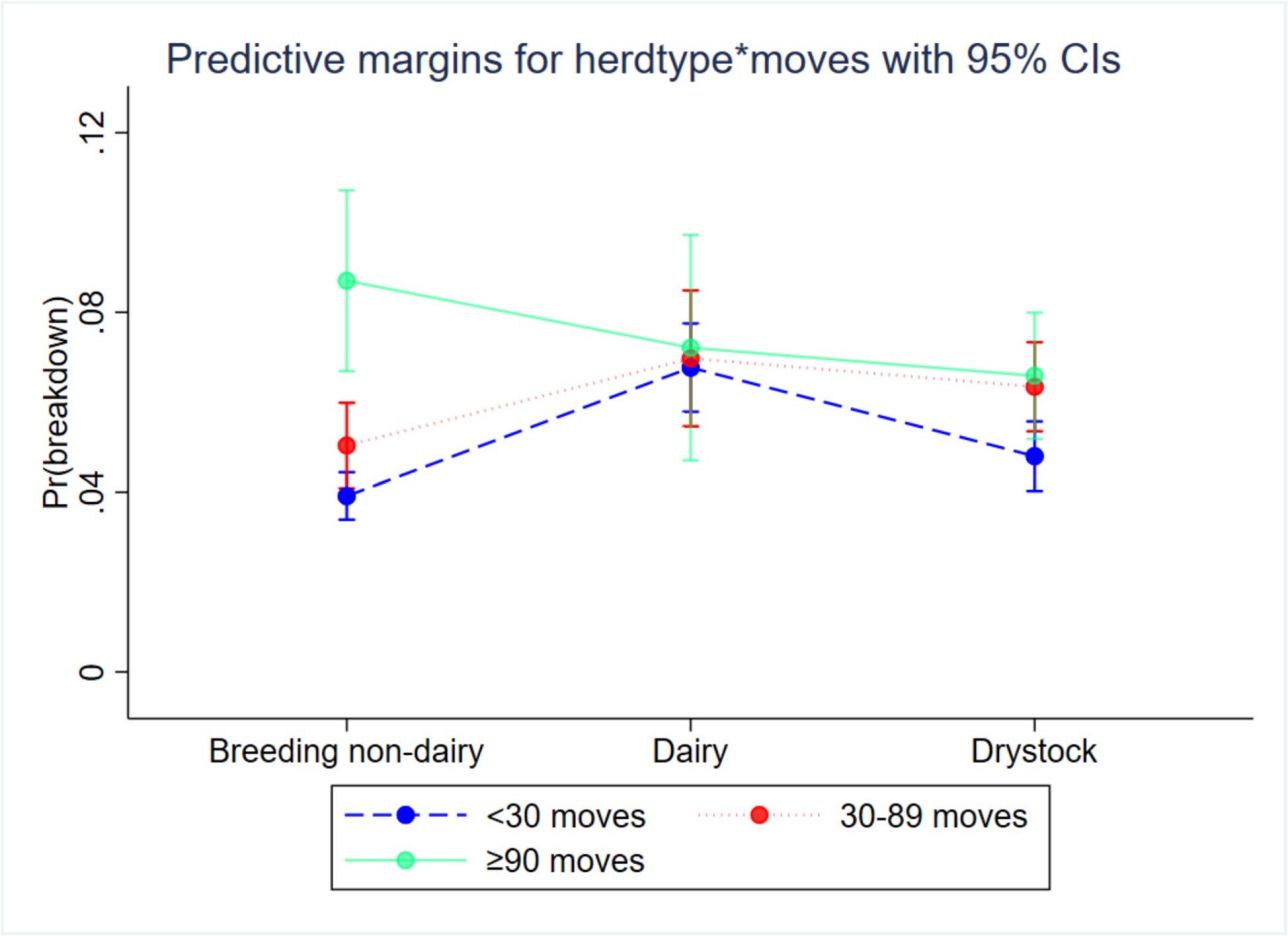
Predictive margins for inward movements (categorised), depending on herd type, on the herd breakdown probability during the risk period on bTB breakdown risk in C10 herds in Ireland

The risk of breakdown increased significantly with herd size. The odds ratio of breakdown occurrence for a one unit increase in log_e_-herd size was statistically significant (OR: 1.547; 95%CI: 1.475–1.622; *P* < 0.001; Fig. 2A). There was evidence that dairy (OR: 1.806; 95%CI: 1.599–2.041; *P* < 0.001) and drystock herds (OR: 1.242; 95%CI: 1.092–1.413; *P* < 0.001) were at significantly increased risk of breakdown, relative to non-dairy breeding herds (Fig. 3), while controlling for covariates. A significant interaction between herd-type and inward movement suggested that movement effects depended on herd-type. In non-dairy breeding herds, there was evidence that increasing the number of movements into the herd was associated with increased breakdown risk, with herds which move in an average of 49 animals (30–89) having 1.308 (95%CI: 1.100–1.557; *P* < 0.001), and herds which move in an average of 165 animals (≥ 90) having 2.395 (95%CI: 1.881- 3.049; *P* < 0.001), times the odds of breakdown relative to herds that moved in an average of 6 animals (< 30) during the risk period (Fig. 3). Similarly, in drystock herds, there were significant increases in risk associated with increasing inward movement (moderate moves: OR:1.354; 95%CI: 1.148–1.597; *p* < 0.001; high moves: OR:1.412; 95%CI: 1.109–1.796; *P* = 0.005), but this was not the case for dairy herds where the overall impact of inward moves was not significant (moderate moves: OR:1.034; *p* = 0.752; high moves: OR:1.073; 95%CI; *P* = 0.704). This was reflected in the interaction term, where there was significant decrease in the effect of movement in dairy relative to non-dairy herds, especially for herds that moved many animals into the herd (OR: 0.448; 95%CI: 0.291–0.960; *P* < 0.001; Fig. 3). It should be noted that only 241 dairy herds (2.99%) were involved in the high inward movement category. Herds that never experienced a breakdown were at significantly decreased risk (OR: 0.742; 95%CI: 0.686–0.803; *P* < 0.001), relative to herds which had one or more breakdowns recorded within the AHCS database (Fig. 2B).

There was no evidence that herds within DEDs wholly (OR: 0.999; 95%CI: 0.892–1.119; *P* = 0.983) or partially (OR: 1.002; 95%CI: 0.918–1.093; *P* = 0.966) assigned to badger vaccination were at significantly increased risk relative to herds found within DEDs without any quartiles assigned to badger vaccination (Fig. 2C). Similar results were found when county has fitted as a fixed effect (Table S3).

## Discussion

Monitoring changing risk profiles across herds is essential when exploring potential drivers of increasing incidence of endemic diseases [14]. Changing patterns in the levels of infection in herds historically known to be of low risk, could be an indicator that aspects of a control programme are performing suboptimally. In the present study, however, hypotheses regarding increased breakdown probability over time for herds with a long history of bTB freedom, and increased bTB risk where such herds were exposed to badger vaccination, were not supported.

Although there was a decrease in the relative risk of a TB breakdown with increasing number of years since a breakdown, the present study demonstrated that herds with long-term official bTB freedom continue to experience some risk of a bTB breakdown, albeit a significantly lesser risk compared to other herds. The present study has shown that in recent years, approximately 3% of C10 status herds break down per annum. Furthermore, the number of C10 herds breaking down over 18 months represents approximately 2.9% of all active herds. However, more importantly, the risk of breakdown is significantly lower for these herds compared to herds with a shorter period of bTB official freedom. These results are consistent with the results of Clegg et al. [12], which found a similar monotonic decline in breakdown probability with increasing years of freedom. In addition, our models have suggested that bTB histories are associated with very long risk “shadows” on herds, with evidence that herds with breakdowns ending more than ten years previously had a slightly increased bTB risk relative to herds with no evidence of breakdown on DAFM records. Again, a very similar result was reported for data in Ireland from 2012 by Clegg et al. [12]. In that paper the odds ratio of TB breakdown in a particular period for herds which had a history of at least one breakdown relative to herds which had no record of a breakdown was 1.24 (95%CI: 1.12–1.37), very similar to the result in the present study (inverse OR: 1.35).

The findings in relation to increased breakdown risk for dairy herds, herds with larger numbers of cattle and herds which buy-in larger numbers of cattle are consistent with previous published work in this area [15–17]. Odds ratio estimates did not straddle 1, with narrow confidence intervals and low p values for each of these covariates, confirm that these herd attributes are likely to have a very substantial bearing on bTB outcomes for an Irish cattle herd during the study period. Indeed, a dominance analysis of risk factors for future bTB breakdown in Ireland identified herd-size, county, and herd-type, respectively, as the most important predictors of future breakdown [18]. The effect of inward movement on bTB risk varied significantly amongst herd types, with a diminished effect for dairy herds (although it should be noted, the cases where dairy herds bought in large numbers of animals were rare and exhibit the widest confidence intervals). Dairy herds had higher overall risk of breakdown, however, non-dairy breeding herds with large numbers of inward movements were typically higher risk than dairy. Given that the threshold we used for a herd to be considered a breeding herd was just one birth, the movement of large numbers of cattle into the non-dairy breeding herds may well be acting as a proxy for activities other than suckler farming. That is, a proportion of our non-dairy breeding herds which buy in large numbers of cattle are likely to be largely focused on finishing and fattening cattle rather than suckling [19]. For such enterprises, biosecurity in terms of buying from a limited number of low-risk herds is likely to be less of a priority than is the case for dairy herds. Some of the cases where dairy herds move in large numbers per year will be a reflection of contract rearing arrangements (i.e. an agreement where a farm owner hires another farmer to raise their livestock, most commonly dairy replacement heifers from a young age until they are close to calving for the first time). While not without their own biosecurity risks, movements of contract reared animals back to the dairy herd can carry less risk than introducing a large number of animals from a myriad of different sources [17] as will be the case with some non-dairy herds with large numbers of inward movements. The risk is likely to be further reduced where the contract rearing relationship is one-to-one, rather than one-to-many or many-to-one. Comparing practices within the dairy sector, large numbers of inward moves to a dairy farm may reflect a farmer preference for contract rearing over, for example, selling of all calves and buying heifers on the point of calving, or recently calved cows. The latter option would also tend to involve buying from multiple sources, a practice that carries with it the risk of “buying in” bTB, even when care is taken as to the risk status of the source herds. For simplicity, we categorised movement into broad groups (low, higher, and very high levels of movement), and therefore there may have been some information loss, which future research could explore more explicitly.

County was found to be a source of variation in the risk of breakdown, as demonstrated by the significant random effect in our model (see supplementary material for predicted variation at county level from our fixed effect model, controlling for covariates). Certain counties were found to have overall higher risks, often corresponding with long term hotspots of infection. For example, County Monaghan has been found to have the highest average relative abundance of badgers across counties, but also has have the highest overall levels of intervention in terms of culling and vaccination [10, 20, 21]. It should be noted, however, that our approach did not account for neighbourhood spatial autocorrelation [22], which could be explored in future analyses.

A secondary hypothesis tested during the present study was examining whether herds in areas designated for badger vaccination were of greater risk of breakdown, relative to herds in DEDs with partial or no badger vaccination designation. Badger vaccination has been perceived as being of lower efficacy by some stakeholders [9], especially relative to culling interventions [23]. Vaccination of badgers as a policy in Ireland was underpinned by laboratory and field studies [5, 24–27], in addition to a non-inferiority trial to assess whether vaccination would perform “no worse” than continued culling [6]. The latter found that in four of seven areas, vaccination performed no worse that culling, but the results were more ambiguous in 2 sites, and a remaining site (Monaghan) showed clear inferiority relative to vaccination. Coupled with this, there was cognisance that badger vaccination would take longer periods than culling to have significant effects on reducing infection pressure [23, 28]. In the present study, there was no association between herd breakdown risk and the landscape scale badger interventions that were occurring in C10 herds. This would suggest that the hypothesis that C10 herds in areas assigned to badger vaccination are at higher risk of bTB breakdown, relative to herds in landscapes with partial or no badger vaccination, should be refuted. However, there was a very small (non-significant in the multivariable models) trend towards increased risk for herds in vaccination areas relative to non-vaccine areas (where culling predominates). Many of the vaccination areas in Ireland are based in good farmland with a high proportion of dairy farms and large herd sizes (often equating to cattle density [29]). Some of these herds also purchase substantial numbers of cattle. Accordingly, in some cases bTB problems in an area may be attributed to the badger vaccination programme, when it is likely that the characteristics of the herds in the area may also be having an effect on bTB outcomes.

There are several implications of this work. Firstly, this analysis highlights that all herd owners should be aware that while having not experienced a breakdown for long periods of up to a decade reduces a herd’s relative risk of breakdown, it does not eliminate the risk. This is especially the case for herds that are engaged in higher risk activities like the introduction of many animals into their herds – indeed, given the worsening epidemiological situation, this risk may intensify as sourcing wholly unexposed stock may become more difficult. There is evidence to suggest that farmers that have not experienced bTB recently are less engaged with implementing biosecurity, for example fencing off wildlife risk areas, than those with more recent experience of bTB [30]. Therefore, it is essential that all farmers irrespective of their risk categories are aware of, and implement, relevant risk mitigation measures.

Addressing the impact of the wildlife management method, there was no evidence that herds within badger vaccination designated areas were at increased, or decreased, risk of bTB breakdown relative to other areas. A model with an interaction between herd type and vaccination treatment also did not support the hypothesis that dairy herds in vaccine areas were at an increased risk of bTB breakdown relative to dairy herds in non-vaccine areas. This refutes the hypothesis that herds in such areas are comparatively at increased exposure, all things being equal, to infection from pathways involving wildlife. However, it is accepted that wildlife interventions can take long periods before measurable impacts in cattle herds are observed [31, 32]. Furthermore, culling interventions can have long term measurable effects for several years, even where the interventions have ceased [10, 31].

### Study limitations

The landscape of Ireland has experienced a mosaic of historic interventions, with differing time periods and histories, which are likely to result in differing badger population densities and levels of infection [7]. We used a very simple classification of local wildlife intervention for this model, namely areas that were *designated* for vaccination, but future work requires better metrics of both wildlife density and infection levels to understand the impacts of current interventions. Designated areas vary widely in the intensity of vaccination applied.

The selection of areas for turnover to vaccination in the past decade was based on two criteria—low badger density (based on previous culling efforts, where repeated culling should have lowered relative abundance significantly; [3]) and low disease prevalence in cattle. Accordingly, while the populations of herds in different badger vaccination categories used in this study appeared roughly similar as regards such attributes as herd type and herd size, the different categories should not be regarded as random assignments- quartiles and areas were assigned to vaccination, or otherwise, based on the epidemiological situation which pertained at a particular point in time. Areas designated as vaccination areas might be expected to have a perceived lower risk going forward, but lower badger density might well have been arrived at through sustained culling in response to a history of bTB problems in an area. Accordingly, the possibility of different background risks, which may have changed over time, and which could go in either direction, for the different vaccination categories cannot be ruled out.

This study focused on C10 herds. Caution would need to be exercised in extrapolating the findings to all herds, even within an Irish context.

### Implications for policy makers

As of 2025, there was broad acceptance that Ireland’s bTB programme needed to be “reset”, in order to get back on track towards eradication. This study informs the debate, by confirming that the assumed lower risk of herds with a longer bTB free history is borne out by recent data. This indicates that policy proposals around integrating risk with trading between herds to lower buyer exposure is supported by evidence. Stakeholders accept that there have been challenges associated with badger vaccination in certain areas of Ireland in recent years. Although not seeking to refute or diminish these issues, this study also informs the discussion around wildlife and bTB, showing that the designation of a local area as regards badger vaccination is not generally a risk factor for bTB breakdown during 2023 to mid-2024, particularly in herds with a long history of being clear of the disease – at least at the scale investigated during this study. Ideally, future studies should attempt to incorporate the mosaic of wildlife interventions and their impacts, but most likely these will require modelling approaches integrated with further empirical analysis [33, 34].

## Supplementary Information

Supplementary Material 1: Figure S1: Number of animals in herds with different designations for badger vaccination- on 31st December 2022. 654 exceptionally large herds with >499 animals are omitted to show the distribution of the majority of herds in more detail. Omitted herds include 233 herds in nonvax DEDs (0.50% of nonvax herds), 294 herds in partvax DEDs (0.73% of partvax herds) and 127 herds in fullvax DEDs (0.67% of fullvax herds). Figure S2: Breakdown of herd types on 31st December 2022 in DED areas with different wildlife protocols. Figure S3: TBHHR on 31st December 2022 for herds in DEDs with different wildlife approaches. Figure S4: Herd size for C10 herds in DEDs with different wildlife approaches- herds with more than 499 animals excluded. Table S1: Summary statistics for number of animals at 31st December 2022 for C10 herds in DEDs with different approaches to bTB in wildlife. Fig S5: Normal plot for county-level residuals from a random effects model of bTB breakdown in Ireland, with county being fitted as a random effect. Figure S6: Predicted marginal probability of C10 herd-breakdown for counties (n=26) within Ireland from a fixed effect logistic model. Table S2: Final random effect model exploring the relationship between breakdown probability for C10 herds in Ireland and the operational assignment of vaccination of badgers at the district electoral division (DED) geographic level. Table S3: Relationship between breakdown probability for C10 herds in Ireland and the operational assignment of vaccination of badgers at the district electoral division (DED) geographic level. Note, county is included as a categorical fixed effect.

## Data Availability

The datasets generated and/or analysed during the current study are not publicly available on privacy grounds but are available from the first author (Michael Horan) on reasonable request.
