# Supplementary material for: Bovine tuberculosis (TB) in herds with long-duration of official freedom during a period of national resurgence of infections
Source: Ir Vet J. 2026 Jan 21;79:13. doi: 10.1186/s13620-026-00330-w (PMC12905887; doi:10.1186/s13620-026-00330-w)
Supplement: Supplementary file 1 — Supplementary Material 1: Figure S1: Number of animals in herds with different designations for badger vaccination- on 31st December 2022. 654 exceptionally large herds with >499 animals are omitted to show the distribution of the majority of herds in more detail. Omitted herds include 233 herds in nonvax DEDs (0.50% of nonvax herds), 294 herds in partvax DEDs (0.73% of partvax herds) and 127 herds in fullvax DEDs (0.67% of fullvax herds). Figure S2: Breakdown of herd types on 31st December 2022 in DED areas with different wildlife protocols. Figure S3: TBHHR on 31st December 2022 for herds in DEDs with different wildlife approaches. Figure S4: Herd size for C10 herds in DEDs with different wildlife approaches- herds with more than 499 animals excluded. Table S1: Summary statistics for number of animals at 31st December 2022 for C10 herds in DEDs with different approaches to bTB in wildlife. Fig S5: Normal plot for county-level residuals from a random effects model of bTB breakdown in Ireland, with county being fitted as a random effect. Figure S6: Predicted marginal probability of C10 herd-breakdown for counties (n=26) within Ireland from a fixed effect logistic model. Table S2: Final random effect model exploring the relationship between breakdown probability for C10 herds in Ireland and the operational assignment of vaccination of badgers at the district electoral division (DED) geographic level. Table S3: Relationship between breakdown probability for C10 herds in Ireland and the operational assignment of vaccination of badgers at the district electoral division (DED) geographic level. Note, county is included as a categorical fixed effect. [file 13620_2026_330_MOESM1_ESM.docx]

# Supplementary material

### Herd size- herds with fewer than 500 animals included


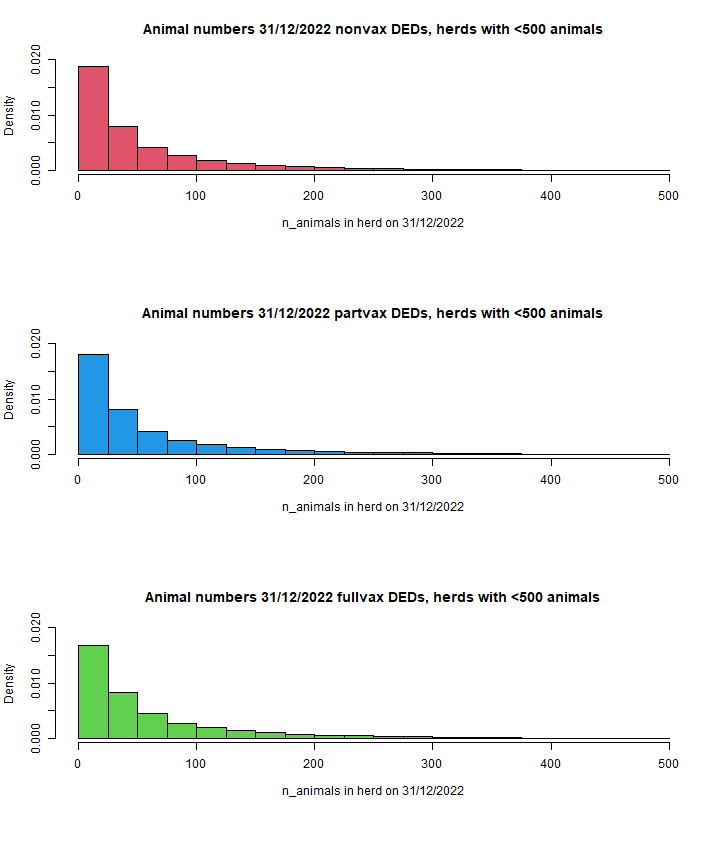


**Figure S1: Number of animals in herds with different designations for badger vaccination- on 31^st^ December 2022. 654 exceptionally large herds with >499 animals are omitted to show the distribution of the majority of herds in more detail. Omitted herds include 233 herds in nonvax DEDs (0.50% of nonvax herds), 294 herds in partvax DEDs (0.73% of partvax herds) and 127 herds in fullvax DEDs (0.67% of fullvax herds).**

### Breakdown of herd types in the different areas

**
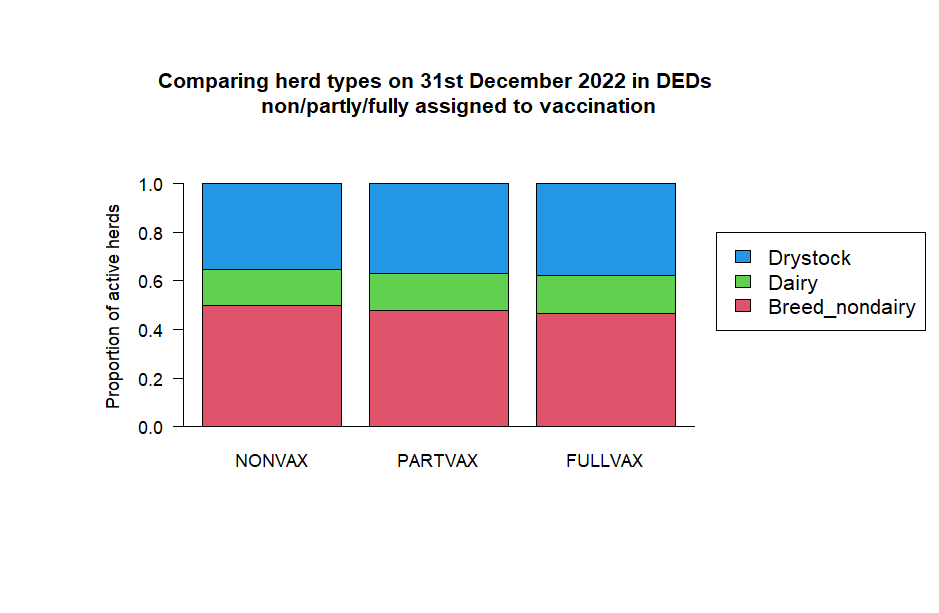
**

**Figure S2: Breakdown of herd types on 31^st^ December 2022 in DED areas with different wildlife protocols.**

### Proportion of herds with different TBHHR statuses in different areas on 31^st^ December 2022


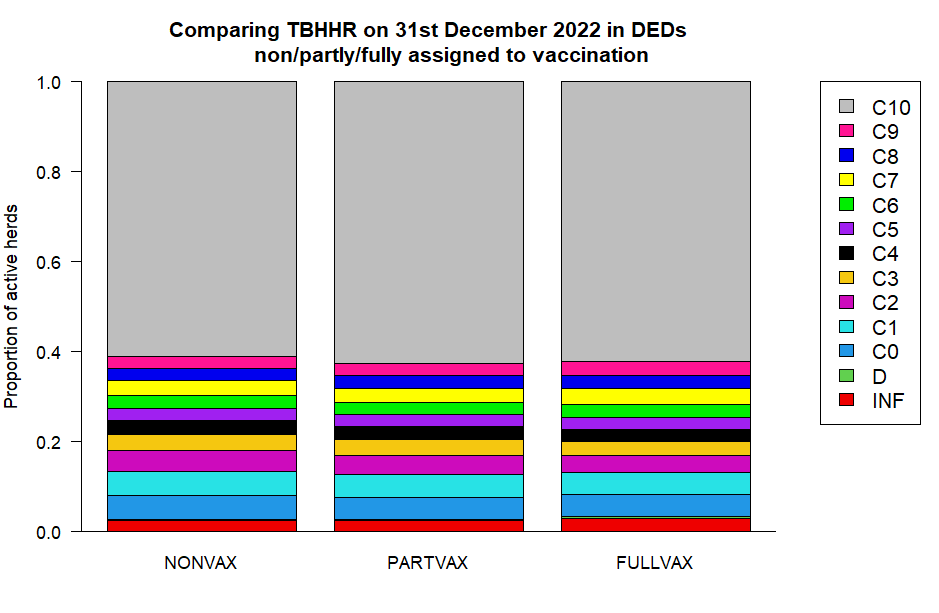


**Figure S3: TBHHR on 31^st^ December 2022 for herds in DEDs with different wildlife approaches**

### Distribution of C10 herd size in different areas on 31/12/2022


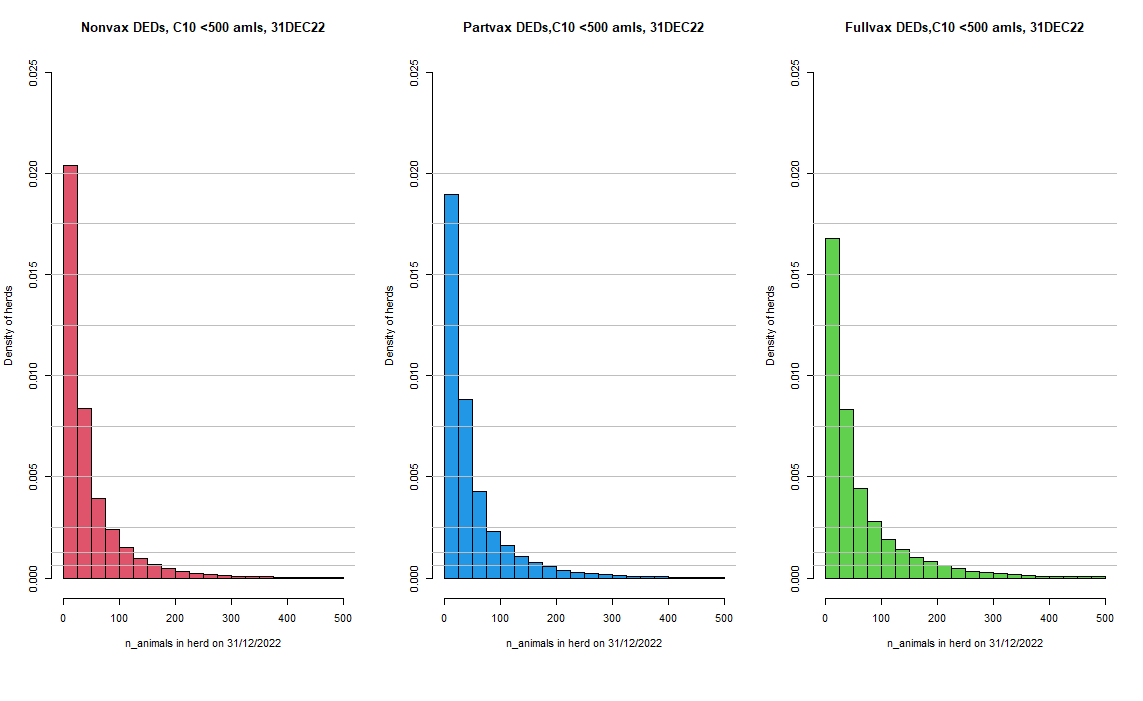


**Figure S4: Herd size for C10 herds in DEDs with different wildlife approaches- herds with more than 499 animals excluded**

**Table S1: Summary statistics for number of animals at 31^st^ December 2022 for C10 herds in DEDs with different approaches to bTB in wildlife.**

### Summary statistics for C10 herd size in different areas on 31/12/2022

| **DED approach** | **Number of herds** | **Min.** | **1st Quartile** | **Median** | **Mean** | **3rd Quartile** | **Max.** |
| --- | --- | --- | --- | --- | --- | --- | --- |
| NONVAX | 28,764 | 0 | 9 | 25 | 46.4 | 57 | 1154 |
| PARTVAX | 25,449 | 0 | 11 | 28 | 51.9 | 62 | 1493 |
| FULLVAX | 11,697 | 0 | 13 | 30 | 54.9 | 67 | 1459 |

**Fig S5: Normal plot for county-level residuals from a random effects model of bTB breakdown in Ireland, with county being fitted as a random effect.**

**Figure S6: Predicted marginal probability of C10 herd-breakdown for counties (n=26) within Ireland from a fixed effect logistic model.**

**Table S2: Final random effect model exploring the relationship between breakdown probability for C10 herds in Ireland and the operational assignment of vaccination of badgers at the district electoral division (DED) geographic level.**

| Parameter | Odds Ratio | P>z | lower 95%CI | upper 95%CI |
| --- | --- | --- | --- | --- |
|  |  |  |  |  |
| Log (herd size) | 1.547 | <0.001 | 1.475 | 1.622 |
|  |  |  |  |  |
| Herd type |  |  |  |  |
| Breeding non-dairy | ref. |  |  |  |
| Dairy | 1.807 | <0.001 | 1.599 | 2.041 |
| Drystock | 1.242 | 0.001 | 1.092 | 1.413 |
|  |  |  |  |  |
| Inward movements (categorised) |  |  |  |  |
| <=30 | ref. |  |  |  |
| 30-89 | 1.308 | 0.002 | 1.100 | 1.557 |
| >=90 | 2.395 | <0.001 | 1.881 | 3.049 |
|  |  |  |  |  |
| Herd type#Inward movements |  |  |  |  |
| Dairy#(30-89 moves) | 0.790 | 0.084 | 0.605 | 1.032 |
| Dairy#(>=90 moves) | 0.448 | <0.001 | 0.291 | 0.690 |
| Drystock#(30-89 moves) | 1.035 | 0.769 | 0.823 | 1.301 |
| Drystock#(>=90 moves) | 0.589 | 0.001 | 0.428 | 0.812 |
|  |  |  |  |  |
| Vaccination exposure |  |  |  |  |
| No vaccination in DED | ref. |  |  |  |
| DED partially vaccinated | 1.002 | 0.966 | 0.918 | 1.094 |
| DED fully vaccinated | 0.999 | 0.983 | 0.892 | 1.119 |
|  |  |  |  |  |
| Historic breakdown status |  |  |  |  |
| Breakdown >10 yrs ago | ref. |  |  |  |
| No recorded breakdowns | 0.742 | <0.001 | 0.686 | 0.803 |
| Constant | 0.010 | <0.001 | 0.008 | 0.013 |
| ρ (intraclass coefficient) | 0.029 | <0.001 | 0.015 | 0.054 |

**Table S3: Relationship between breakdown probability for C10 herds in Ireland and the operational assignment of vaccination of badgers at the district electoral division (DED) geographic level. Note, county is included as a categorical fixed effect.**

| Parameter | Odds Ratio | P>z | lower 95%CI | upper 95%CI |
| --- | --- | --- | --- | --- |
|  |  |  |  |  |
| Log (herd size) | 1.547 | <0.001 | 1.475 | 1.623 |
|  |  |  |  |  |
| Herd type |  |  |  |  |
| Breeding non-dairy | ref. |  |  |  |
| Dairy | 1.821 | <0.001 | 1.611 | 2.058 |
| Drystock | 1.242 | 0.001 | 1.091 | 1.414 |
|  |  |  |  |  |
| Inward movements (categorised) |  |  |  |  |
| <=30 | ref. |  |  |  |
| 30-89 | 1.312 | 0.002 | 1.102 | 1.561 |
| >=90 | 2.405 | <0.001 | 1.889 | 3.063 |
|  |  |  |  |  |
| Herd type#Inward movements |  |  |  |  |
| Dairy#(30-89 moves) | 0.785 | 0.076 | 0.600 | 1.025 |
| Dairy#(>=90 moves) | 0.448 | <0.001 | 0.291 | 0.690 |
| Drystock#(30-89 moves) | 1.035 | 0.768 | 0.823 | 1.301 |
| Drystock#(>=90 moves) | 0.590 | 0.001 | 0.428 | 0.814 |
|  |  |  |  |  |
| Vaccination exposure |  |  |  |  |
| No vaccination in DED | ref. |  |  |  |
| DED partially vaccinated | 1.007 | 0.884 | 0.921 | 1.100 |
| DED fully vaccinated | 0.997 | 0.955 | 0.888 | 1.119 |
|  |  |  |  |  |
| Historic breakdown status |  |  |  |  |
| Breakdown >10 yrs ago | ref. |  |  |  |
| No recorded breakdowns | 0.743 | <0.001 | 0.686 | 0.804 |
|  |  |  |  |  |
| County |  |  |  |  |
| Carlow | ref. |  |  |  |
| Cavan | 1.705 | 0.014 | 1.112 | 2.615 |
| Clare | 1.515 | 0.056 | 0.989 | 2.320 |
| Cork | 1.722 | 0.008 | 1.155 | 2.567 |
| Donegal | 1.572 | 0.040 | 1.020 | 2.422 |
| Dublin | 3.951 | <0.001 | 2.011 | 7.760 |
| Galway | 1.166 | 0.465 | 0.773 | 1.759 |
| Kerry | 1.686 | 0.013 | 1.116 | 2.547 |
| Kildare | 2.355 | <0.001 | 1.484 | 3.737 |
| Kilkenny | 1.583 | 0.033 | 1.037 | 2.417 |
| Laois | 1.827 | 0.006 | 1.185 | 2.817 |
| Leitrim | 1.930 | 0.005 | 1.224 | 3.041 |
| Limerick | 1.168 | 0.467 | 0.768 | 1.775 |
| Longford | 3.015 | 0.000 | 1.959 | 4.639 |
| Louth | 2.005 | 0.008 | 1.195 | 3.365 |
| Mayo | 1.058 | 0.795 | 0.694 | 1.612 |
| Meath | 2.233 | 0.000 | 1.448 | 3.444 |
| Monaghan | 2.389 | 0.000 | 1.562 | 3.654 |
| Offaly | 1.361 | 0.179 | 0.869 | 2.133 |
| Roscommon | 1.742 | 0.010 | 1.141 | 2.658 |
| Sligo | 1.896 | 0.005 | 1.213 | 2.964 |
| Tipperary | 1.531 | 0.040 | 1.019 | 2.301 |
| Waterford | 0.898 | 0.651 | 0.565 | 1.429 |
| Westmeath | 1.449 | 0.118 | 0.910 | 2.308 |
| Wexford | 1.353 | 0.181 | 0.869 | 2.106 |
| Wicklow | 3.898 | <0.001 | 2.435 | 6.239 |
| Constant | 0.006 | <0.001 | 0.004 | 0.009 |
